# Small Animal MRI

**Published:** 1995

**Authors:** Margaret Acara, James Alletto, Cynthia Dlugos, Roberta Pentney

**Affiliations:** Margaret Acara, Ph.D., is a professor in the Department of Pharmacology and Toxicology, School of Medicine and Biomedical Sciences, State University of New York, Buffalo, New York. James Alletto, Ph.D., is a postdoctoral fellow in the Biophysics Department, Medical College of Wisconsin. Cynthia Dlugos, Ph.D., is a clinical assistant professor and Roberta Pentney, Ph.D., is a professor in the Department of Anatomy and Cell Biology, School of Medicine and Biomedical Sciences, State University of New York, Buffalo, New York

**Keywords:** animal model, magnetic resonance imaging, thiamine deficiency, brain damage, AOD abstinence, AODE (alcohol and other drug effects)

Alcoholism and other diseases affect the structure and function of many organs in the human body, including the brain. To understand the causes and to diagnose and track the progress of such diseases, scientists must understand the structural and functional changes in the affected organs. For many studies, particularly those involving the brain, researchers rely on non-invasive methods to visualize the organ under investigation. One technique commonly used to obtain images of a patient’s brain or other area of the body is magnetic resonance imaging (MRI). This technique exposes the subject to radio waves in the presence of a strong magnetic field and then measures how the atoms that make up the body’s tissues absorb the radio waves’ energy. The manner of absorption varies, depending on the composition of the tissues (e.g., their water or fat content); with the help of computers, these variations can be converted into images.

This article describes how MRI technology can be used not only on human patients but also in studies of laboratory animals that frequently serve as experimental models for human diseases. Also presented are findings of MRI studies that analyzed the effects of thiamine deficiency—the lack of a vitamin required for normal brain functioning—on the brain structure of rats. Preliminary findings indicate that similar studies can be used to assess the effects of long-term alcohol consumption and withdrawal on the brain.

Although MRI has been used successfully in humans to diagnose diseases and study alterations in organ structures, its applications in elucidating the cause of a pathology or functional abnormality are more limited. To investigate the cause of a disease, researchers ideally would compare images of patients taken before and after they develop the disorder. Only rarely, however, do patients have images available for comparison that were taken prior to an illness. Alternatively, investigators can compare one group of subjects with a disease (e.g., alcoholics) with another group of people without the disease (e.g., nonalcoholics) to identify consistent differences between the two groups that might shed light on the cause of alcoholism. The subjects in such studies, however, will have different characteristics in addition to their alcohol intake (e.g., their medical and drinking histories and their genetic makeup). To make valid observations and to control for all possible variations among subjects, researchers must examine a large number of patients before attributing specific differences between the two study groups to alcohol use.

Because of these limitations of human studies, researchers frequently rely on animal models, often using rats and other small animals. In general, animal studies provide more consistent information with fewer subjects than do human studies. The animals can be examined both before and after they develop a certain disease or undergo a particular treatment. Furthermore, animal studies can be rigidly controlled so that a particular change in organ structure or function can be directly attributed to the modification of a single experimental variable. Differences between animals can be minimized by using animals from one family (i.e., strain), a method that in humans would be feasible only with identical twins. And in contrast to studies on humans, studies using animals can be conducted across an animal’s life span (about 2.5 years for rats), allowing the investigation of age-related effects.

## Conventional Techniques

Histological techniques (i.e., studies that determine the structure of organs by examining thin tissue slices under a microscope) are commonly used to follow disease development in animals. Although these techniques allow researchers to view directly the tissue under investigation, they have several drawbacks, especially in studies of the brain.

First, the animals must be euthanized to remove small pieces of tissue for preparation before examination. Second, constraints of both time and expense generally restrict such studies to only one tissue and a few experimental parameters; consequently, the analyses could fail to detect the full effects of a disease or experimental procedure.

Third, because the animals are euthanized to perform the measurement, each animal can be assessed only at a single timepoint during disease. To examine the entire disease process, researchers must study multiple animals at different stages of disease development and thus must estimate the best timeframe for examining each animal. Because animals can vary significantly in the rate of disease progress, these estimates may miss their mark. For example, one rat may reach a particular stage of a disease 2 weeks earlier than another rat, leading to wide variability in data.

To eliminate some of these problems and to take full advantage of the potential of animal studies, scientists have adapted noninvasive techniques used in humans, such as MRI, for use in small animals (Henkelman et al. 1987; Fiel and Button 1990; Ballon et al. 1989; Yang et al. 1996). Small animal MRI (SAMRI) technology now has become so refined that it can produce very detailed images of an animal’s brain or other organs.

## Contributions of SAMRI

Like MRI in humans, SAMRI is a noninvasive technique. Its primary advantage over other procedures is that it is performed in living animals and therefore can be repeated several times in the same animal to track a disease’s progress. Experiments that previously required hundreds of animals now may only use a dozen. Other advantages of this technique include: Comparable stages of disease progression can be pinpointed among several animals; tissue changes can be viewed as a dynamic process, exactly as they appear in a living animal; three-dimensional relationships in tissue structures that are not evident in histological studies may be examined; researchers can examine more than one tissue simultaneously and thus detect and analyze concurrent and/or related changes in several tissues; and computerized image analysis allows researchers to measure quantitatively the tissues of interest.

## How Is SAMRI Done?

**Figure 1:**
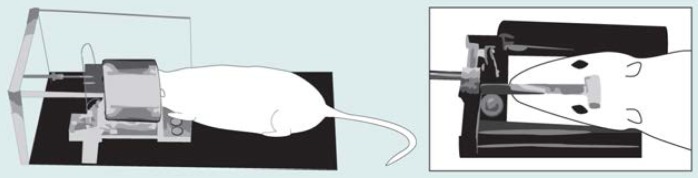
The small animal magnetic resonance imaging apparatus. To ensure precise and reproducible positioning, the rat is lightly anesthetized before being placed in the head brace. The head brace is surrounded by a metal coil that generates the magnetic field and the radiofrequency waves used to create the brain image. The plastic bar (see inset) on the rat’s head is one of the three markers used to align images taken at different times.

Adapting human MRI to small animals was not an easy process. Consider, for example, the much greater power of resolution necessary to achieve the same precision in defining structures in the brain of a rat as in the much larger brain of a human. Recent technological advances, however, provide SAMRI images with a resolution equal to that obtained in MRI’s of the human brain and on par with the detail seen in histological sections. For example, the sharpness, and thus the resolution, of an image depends in part on the geometric relationship between the object being imaged and the MRI chamber housing it. This chamber contains a coil that generates the magnetic field to which the subject is exposed. Special coils have been designed to perform MRI’s on specific regions of the human body (e.g., the arm). These smaller coils also have been used in some animal imaging studies (Fiel and Button 1990). To obtain optimal images of organs, such as the rat brain, however, coils must be custom designed (figure 1). The coils then can be tuned to the magnetic field of a clinical scanner (Pentney et al. 1993).

Another adaptation of SAMRI was the designing of a head brace to orient and secure the rat’s head within the SAMRI apparatus (Pentney et al. 1993). Precise positioning is necessary so that researchers can compare various images taken of the same rat at different times or of different rats. The three-dimensional (i.e., stereotactic) head brace incorporates three markers, which are aligned to certain anatomical parts of the rat (i.e., the teeth, ears, and top of the skull) and thus allow duplication of the cross-section (i.e., plane) of the brain being imaged. Computers convert the data into images that represent sequential 0.7 mm-thick sections through the brain. The images of these sections then can be compiled to visualize the entire brain.

## SAMRI and Thiamine

Thiamine, or vitamin B_1_, is essential for normal brain metabolism and, in turn, normal brain functioning. Several enzymes involved in the brain’s metabolism of glucose—the body’s primary energy source—require thiamine to function (Butterworth 1989; Butterworth et al. 1993; Langlais 1995). Thiamine deficiency has been associated with severe cognitive dysfunction, most prominently with Wernicke-Korsakoff syndrome (WKS), a disorder characterized by the inability to learn and remember new information for more than a few seconds (i.e., anterograde amnesia) and other cognitive deficits (Langlais 1995).

Thiamine deficiency is relatively common among alcoholics, and the majority of patients diagnosed with WKS are alcohol dependent (for a review, see Langlais 1995). Alcoholism may contribute to thiamine deficiency in several ways. For example, poor nutrition, which is common in alcoholics, may result in insufficient thiamine intake. Furthermore, alcohol may interfere with thiamine absorption in the intestines (Butterworth 1989). Finally, alcohol may contribute to increased thiamine excretion in the urine (Acara et al. 1983). Thus, researchers are studying the effects of thiamine deficiency on brain structure and function to better understand alcohol’s effects on the brain and the mechanisms involved in the development of WKS.

Rats often are used as a model to study the consequences of both thiamine deficiency and alcohol abuse. To validate the rat model, however, researchers first must duplicate observations made in human alcoholics using studies on the animals. For example, several studies with human subjects, including WKS patients, have demonstrated certain brain changes associated with alcohol abuse (Pfefferbaum et al. 1992; Shear et al. 1994). These changes include the enlargement of cavities (i.e., ventricles) in the brain that produce cerebrospinal fluid. Increased ventricle size usually results from a concomitant loss of brain tissue (Pfefferbaum et al. 1992).

**Figure 2:**
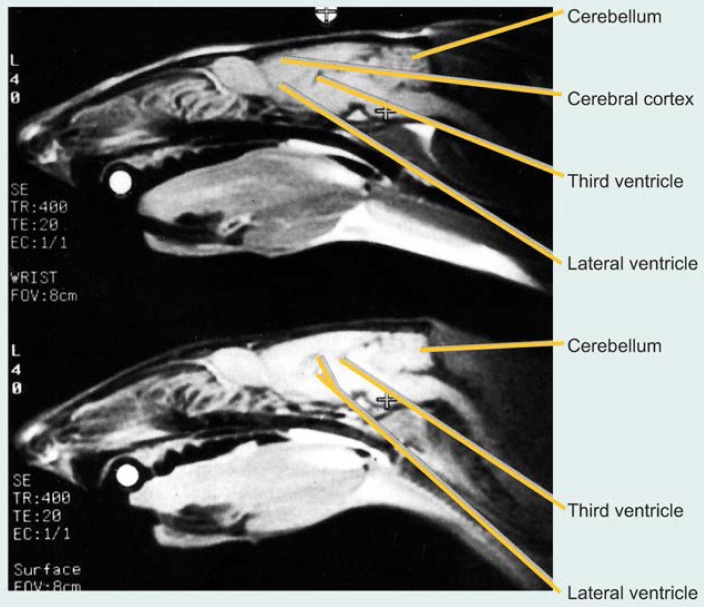
Small animal magnetic resonance images showing the effects of thiamine deficiency on the size of fluid-filled brain cavities (ventricles) in the rat. The images are of a lengthwise cross-section of the brain.^1^ The upper image shows a rat before the start of a thiamine-deficient diet. The lower image is of the same rat after 6 weeks on the diet and shows enlargement of the lateral and third ventricles as well as a loss of fatty tissue and muscles in the rat’s neck region. The crosses and white circles represent three anatomical markers located near the front teeth (incisors), ears, and the top of the skull (bregma) to allow accurate comparison of images taken at different times. SOURCE: Adapted from Pentney, R.J.; Alletto, J.J.; Acara, M.A.; Dlugos, C.A.; and Fiel, R.J. Small animal magnetic resonance imaging: A means of studying the development of structural pathologies in the rat brain. *Alcoholism: Clinical and Experimental Research* 17(6): 1301–1308,1993.

Pentney and colleagues (1993) investigated whether experimental thiamine deficiency in rats could result in similar changes in brain structure and, if so, whether SAMRI could detect these changes. Thiamine deficiency was induced in male rats by feeding them a thiamine-deficient diet for approximately 6 weeks. Control rats received a standard diet containing adequate thiamine levels. Brain images of both groups were generated before and at selected times during the experiment to compare the images taken from the same rat before and after the study period. Images of a lengthwise section separating the right and left sides of the brain demonstrated that after 6 weeks of thiamine deficiency, the rats’ brain ventricles were significantly larger than at the beginning of the experiment (figure 2), whereas the control animals showed no significant changes during the same period. Thiamine deficiency also resulted in extensive loss of fatty tissue and muscle in the rats’ head and neck regions. This loss, which was observed in all thiamine-deficient animals but not in the control animals, likely is attributable to inadequate glucose metabolism, which leads to lower energy production and reduced formation of fat tissue (Butterworth et al. 1993).

**Figure 3:**
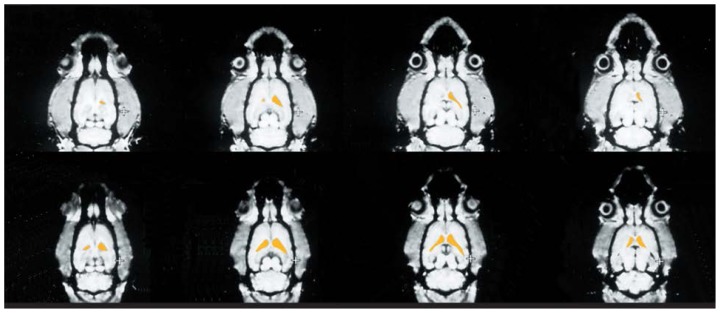
Small animal magnetic resonance images of a rat brain before and after thiamine deficiency. The images represent horizontal cross-sections progressing from the top of the head (left images) to the base of the skull (right images). The images on the top show the rat’s brain before the experiment; those on the bottom were obtained after the rat received a thiamine-deficient diet for 6 weeks. In each of the cross-sections analyzed, the ventricles (indicated in yellow) were larger after thiamine deficiency than before the experiment. SOURCE: Adapted from Pentney. R.J.; Alletto, J.J.; Acara, M.A.; Dlugos, C.A.; and Fiel, R.J. Small animal magnetic resonance imaging: A means of studying the development of structural pathologies in the rat brain. *Alcoholism: Clinical and Experimental Research* 17(6): 1301–1308.1993.

The increase in ventricle size induced by thiamine deficiency was even more obvious in a series of images of horizontal cross-sections of a rat’s brain from the top of the head to the base of the skull (figure 3). For each cross-section analyzed, the ventricles were larger after induced thiamine deficiency than before. Based on such contiguous images, computerized integration of the areas of interest (e.g., the ventricles) eventually will allow researchers to determine the volumes of these structures. Thus, SAMRI of rats confirmed earlier observations of human WKS patients that thiamine deficiency could induce changes in brain structures, including enlargement of the ventricles and the associated loss of brain tissue.

**Figure 4:**
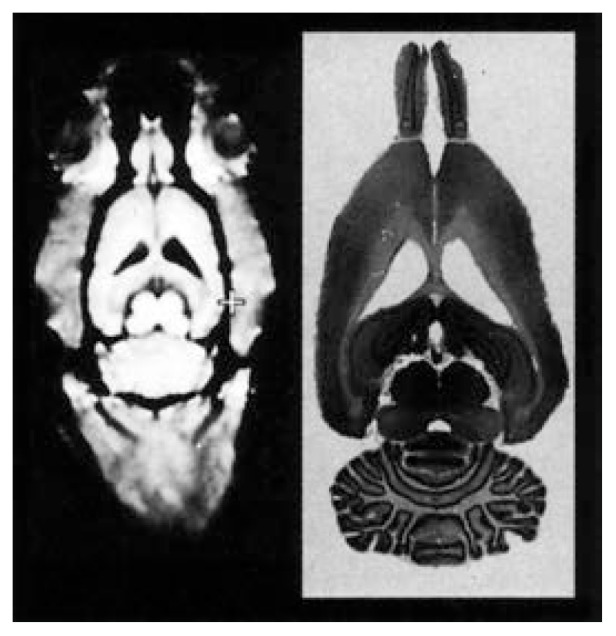
Comparison of a small animal magnetic resonance (SAMRI) image (left) and the histological examination of the corresponding brain slice (right). The SAMRI image shows the brain as well as the surrounding skull. All the brain structures that can be seen in the histological slice are visible in the corresponding image. SOURCE: Adapted from Pentney et al. 1993.

The SAMRI images of thiamine-deficient rats also were compared with histological brain slices obtained from the same animals to determine whether SAMRI was as sensitive as histological examination in detecting morphological changes in the brain. At the end of the 6-week experiment, 0.1 mm-thick histological brain sections were prepared from each rat and compared with the final SAMRI image obtained from the same rat (figure 4). This comparison showed that all the thiamine deficiency-induced changes detectable in the histological section (e.g., the degree of increase of the ventricles) also were visible in the SAMRI image. Thus, the noninvasive SAMRI technology is a powerful and useful substitute for histological examinations.

## SAMRI and Alcohol-Induced Changes

As previously noted, alcohol researchers study the effects of thiamine deficiency because it frequently occurs in alcoholics and may contribute to their brain damage and neurological dysfunction (e.g., WKS). Moreover, some researchers have detected increased amounts of fluid in the brains of human alcoholics (Chick et al. 1989), a change that could indicate enlarged ventricles comparable to those observed in thiamine-deficient subjects. Based on the findings that SAMRI can detect ventricle enlargement in the brains of thiamine-deficient rats, this technique is now being used to study the effects of chronic alcohol consumption on rat brain morphology.

In these studies, the rats receive a solution of 20-percent alcohol and 80-percent tap water as their only fluid source, and the control rats receive plain tap water. Over several months, the researchers take images of the animals’ brains to determine if any morphological changes have occurred in the brains of the alcohol-consuming rats compared with the control rats. Preliminary findings suggest, for example, that the ventricles are enlarged after 9 months of alcohol intake but still maintain their normal sizes after 6 months of alcohol intake. More experiments are necessary for researchers to compare adequately alcohol-consuming animals with control animals and to verify that any observed differences directly result from alcohol intake and not from the animals’ diet, malnutrition, or other similar factors. Nonetheless, SAMRI provides a tool for researchers to track the effects of long-term alcohol consumption under controlled conditions using an animal model. The findings of such studies will help elucidate the consequences of chronic alcohol consumption in humans.

New studies also are using SAMRI to investigate the effects of alcohol withdrawal and prolonged abstinence after long-term alcohol consumption. These studies will shed light on the extent to which alcohol-induced changes in brain morphology are reversible. Preliminary findings indicate that enlarged ventricles observed in alcohol-consuming rats may return to their original size within 6 months after the withdrawal of alcohol.

Finally, SAMRI also permits the examination of alcohol’s effects on tissues other than the brain. For example, in preliminary studies of ventricular enlargement in the alcohol-consuming animals, the rats exhibited wasting of muscle and fat tissue similar to that observed in the thiamine-deficient animals. Similarly, scientists will be able to use SAMRI to study alcohol-induced changes in brain structures other than the ventricles (e.g., different areas of the cortex) and the relative abundance of gray and white matter in the brain. Thus, future studies can be designed to take ample advantage of the multiplicity of information gathered through imaging of the whole animal.

## Conclusions

The studies of the brains of thiamine-deficient or alcohol-consuming rats described above illustrate the potential of SAMRI for determining the consequences of chronic alcohol consumption and for potentially elucidating some of the causes of alcoholism. Many of these analyses cannot be conducted in humans because the experimental conditions (e.g., the amount and duration of alcohol consumption or nutritional intake) cannot be controlled adequately. Furthermore, experiments that track a disease’s progress and thus take a few weeks or months to conduct in rats would require years to conduct in humans. The data from animals offer important insights into some of the causes and consequences of alcoholism and eventually may lead to new diagnostic, prevention, and treatment approaches in humans.

In addition to providing structural information about various tissues by generating images with a clarity comparable to histological sections, MRI technology can be applied to studies of chemical and biochemical processes and organ functions. Magnetic resonance spectroscopic imaging (MRSI) uses the same principle as MRI to determine the metabolic activity of tissues, including the brain. Combining imaging technologies such as SAMRI with analytical MRSI could offer new analytical research tools. For example, SAMRI could generate an image of a particular tissue in a living animal while MRSI simultaneously identifies and measures biochemical compounds and processes and thus determines the rates of metabolic changes. Such analyses would reduce the time needed to conduct many experiments as well as improve the quality of the experiments conducted.

The potential applications of magnetic resonance technology are numerous and can benefit many biomedical investigations. For example, using animal models, researchers can track the biochemical, pathological, and physiological variables of a disease throughout the disease process. They also can evaluate the efficacy of different medications and their specific effects on each variable during the course of a disease. Such studies eventually will lead to the development of better medications and treatment regimens for diseases in humans.
